# Using electrooculography with visual stimulus tracking test in diagnosing of ADHD: findings from machine learning algorithms

**DOI:** 10.55730/1300-0144.5502

**Published:** 2022-06-13

**Authors:** Fatma LATİFOĞLU, Mustafa Yasin ESAS, Ramis İLERİ, Sevgi ÖZMEN, Esra DEMİRCİ

**Affiliations:** 1 Department of Biomedical Engineering, Engineering Faculty, Erciyes University, Kayseri, Turkey; 2 Department of Child and Adolescent Psychiatry, Erciyes University School of Medicine, Kayseri, Turkey

**Keywords:** Signal processing, electrooculography, visual stimulus, attention deficit hyperactivity disorder, classification

## Abstract

**Background/aim:**

Attention deficit hyperactivity disorder (ADHD), one of the most common neurodevelopmental disorders in childhood, is diagnosed clinically by assessing the symptoms of inattention, hyperactivity, and impulsivity. Also, there are limited objective assessment tools to support the diagnosis. Thus, in this study, a new electrooculography (EOG) based on visual stimulus tracking to support the diagnosis of ADHD was proposed.

**Materials and methods:**

Reference stimulus one-to-one tracking numbers (RSOT) and colour game detection (CGD) were applied to 53 medication-free children with ADHD and 36 healthy controls (HCs). Also, the test was applied six months after the treatment to children with ADHD. Parameters obtained during the visual stimulus tracking test were analyzed and Higuchi fractal dimension (HFD) and Hjorth parameters were calculated for all EOG records.

**Results:**

The average test success rate was higher in HCs than in children with ADHD. Based on machine learning algorithms, the proposed system can distinguish drug-free ADHD patients from HCs with an 89.13% classification performance and also distinguish drug-free children from treated children with an 80.47% classification performance.

**Conclusion:**

The findings showed that the proposed system could be helpful to support the diagnosis of ADHD and the follow-up of the treatment.

## 1. Introduction

Attention deficit hyperactivity disorder (ADHD) is a neurodevelopmental disorder that typically appears in early childhood, before the age of twelve [1]. According to a meta-analysis of 19 studies with over 55,000 participants, 5.9% of youth fulfill the diagnostic criteria for ADHD [2]. Another meta-analysis revealed no significant variations in prevalence across North America and Europe, Asia, Africa, South America, and Oceania, based on 135 research studies including a quarter-million young people [3]. A meta-analysis of 20 studies involving over 26,000 participants discovered that 2.8% of people meet ADHD criteria [4]. ADHD is associated with various impairments such as low school performance and academic achievement, poor social relations with peers and family, increased aggression, risky behaviors, early substance experimentation/abuse, as well as internalizing and/or externalizing comorbidities [5].

There is no specific test for the diagnosis of ADHD. Clinicians diagnose ADHD based on symptoms of inattention, hyperactivity, and impulsivity and diagnostic procedures include the following: the assessment of DSM-V ADHD criteria, a medical examination, information from school, interviews with family members, ADHD rating scales such as the Conners Rating Scale for both parents and teachers, and neuropsychological tests. Psychometric tests could be used to support the diagnosis of ADHD, but they are not only specific to the diagnosis of ADHD [6–10]. On the other hand, there are limited objective assessment tools to support diagnosis and eliminate or minimize subjectivity.

Electrooculography (EOG) is a technique to record the electrical activity of eye movements, using surface electrodes. An EOG offers crucial information on electrical activity in the eye and also provides useful data for researchers since it can be used for the detection of eye movements, visual disturbances, and sleep status [11,12]. In the literature on eye tracking, systems using video images, infrared light detection, and EOG signals have been developed. Different techniques such as Hough transformation, Kalman filtering, biometric recognition, and “Likely Hood” modeling have used video images for eye-tracking systems [13–16]. On the other hand, there are limited studies based on eye movement in the literature on ADHD diagnosis. In one study, saccadic eye movement deficits in adults with ADHD were reported [17]. Tsang et al. reported that visual attention was reduced in children with ADHD and the duration of social fixation was shorter compared to healthy subjects [18]. Marotta et al. investigated interpersonal and social problems seen in ADHD and reported problems in right-left eye follow-up and eye fixation, especially with social stimuli [19]. Another study on dynamic eye movement and pupil changes, behavioral changes, and their relationships with neuropsychological test scores could be useful in the diagnosis of ADHD [20]. There are still limited studies on the follow-up of eye movements through video systems due to the high cost of eye-tracking devices and the complexity of video analysis, considering the computational processing. Also, contact lenses, glasses, and iris color have a negative impact on the eye-tracking camera. As an alternative method to eye-tracking devices, eye monitoring systems using EOG signals could be more practical and have a lower cost. In this study, we aimed to develop an inexpensive eye-tracking system and extract attributes that can support the diagnosis of ADHD. Our preliminary studies on this subject were conducted with a limited number of participants using different analysis methods and visual stimulus tests. According to these studies, the accuracy rate of the following stimulus in the direction of movement for children with ADHD was lower, compared to the reference movement [21,22]. In this recent study, EOG signals were recorded using a BIOPAC system with a newly developed visual stimulus test, and the obtained signals were analyzed with machine learning algorithms. Therefore, we have presented a new tool to help ADHD diagnosis, with a novel visual stimulus tracking test using EOG signals, based on the experiences from our preliminary study. In this proposed system, EOG signals were obtained with a new visual stimulus tracking test, based on associated with selective attention processing speed, and performance in different follow-up conditions including visual and auditory distractors. Considering the studies on eye movements in the literature, we thought that the classification of EOG signals could support the clinical diagnosis of ADHD. Thus, we obtained EOG signals by using signal processing methods to use these features to help physicians in the diagnosis and follow-up of ADHD.

## 2. Materials and methods

### 2.1. Participants

Sixty drug-free children aged 8–13 with a diagnosis of ADHD according to DSM-5 were included in the study. All children in the study were recruited in Erciyes University Faculty of Medicine, Child and Adolescent Psychiatry Outpatient Clinic, and were evaluated with KD-SADS-PL [23]. Children and adolescents with comorbid psychiatric diagnoses, mental retardation, neurological, metabolic, and endocrinological diseases were excluded. The healthy control group (HCs) consisted of 36 children and adolescents who do not have mental retardation, any psychiatric disorder, or chronic disease. Participants who had a hearing and/or visual problems and also used any drug that could affect the central nervous system (CNS) were also excluded.

The treatment of the patients was determined independently of the clinicians who designed the study. The patients were selected from drug-free children who would receive OROS- Methylphenidate (MPH) treatment. Response to treatment was also evaluated 2 months after treatment onset. The patients were divided into two subgroups: responders and nonresponders, and children who were adherent to treatment were excluded from the analysis. Finally, data of 53 children were included.

This study was approved by the local ethical committee of the university (2015/90) and the procedures were according to the ethical standards of the responsible committee on human experimentation. Written informed consent was obtained from both children and their parents.

### 2.2. Power analysis

A power analysis was performed. The Type-I error rate was 0.05 and the Type-II error rate was 0.020 in the power analysis. The CDG parameter was utilized for power analysis, in order to establish the sample size since the CGD parameter has the highest success in ADHD/healthy classification (CDG success percentages; HCs:39.02, ADHD:4.62). When the power was considered 80%, the required minimum number of subjects in each group was calculated as 19 for the recent study. In addition, when other parameters were considered, the required minimum number of subjects in each group was calculated as 31.

### 2.3. EOG records

First records were obtained from 53 children with ADHD and 36 HCs. Six months after treatment, the second set of records was obtained from 39 patients who responded to the treatment. Therefore, a total of 128 EOG records were analyzed to determine the diagnostic and treatment status.

#### 2.3.1. Visual stimulus tracking test

**Figure 1:**
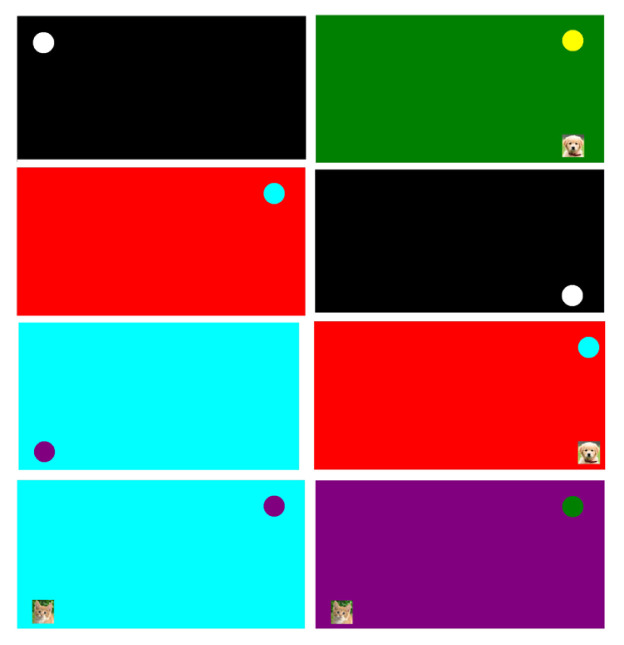
Visual stimulus tracking test.

The developed visual stimulus tracking test provided the opportunity to examine horizontal and vertical eye movements under stimuli with visual and auditory distortion. The visual stimulus tracking test was prepared with C# programming language and in front of a 24” monitor, 30 cm from the screen, the eye level of the individual was set at the same level as the midpoint of the screen, as seen in Figure 1. The test program started with the tracking of white visual stimuli on a black background after calibration and continued with the 10 visual successive stimuli in the background with different colors and the different background colors associated with a particular pattern. The test consisted of 3 stages; in the first stage, only visual stimuli were used moving in accordance with square and z shapes on the screen. The same test was repeated 10 times and lasted a total of 55 s. In the second stage of the test, also visual distractors were added to the initial procedures. Cat and dog pictures appeared suddenly at different points from the stimulus. The second stage of the test lasted for 60 s, followed by the third stage of the test. The third part of the test was identical to the second part but also included auditory distractors. The third part of the test was 60 s.

EOG recordings were obtained simultaneously while children watched visual stimuli that appear on the monitor during the test. The test was carried out for a total of about 3 min.

#### 2.3.2. EOG recording system

The BIOPAC-MP150 developed by Biopac Systems Inc. was used for the recording of EOG signals (www.biopac.com/wp-content/uploads/MP150-Systems.pdf). Two EOG 100C EOG modules compatible with the MP150 physiological signal recorder were used during the test. Thus, a two-channel EOG recording was used. EOG signals were obtained via electrical potential change on noninvasive surface electrodes attached to the right, left, upper, and lower regions of the eye, based on the reference electrode. Noninvasive Ag-AgCl surface electrodes were used during the measurements. Initially, EOG records were obtained from patients with ADHD. Subsequently, after 6 months of drug treatment, the second EOG signals were obtained from the same patient group. In addition, records were obtained from HCs. The obtained EOG signals were recorded for two channels in “.txt” format, processed in MATLAB, and analyzed.

#### 2.3.3. Signal processing studies

**Figure 2:**
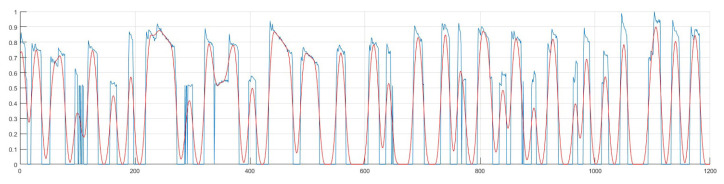
An example of EOG signals (above) and reference stimulus signal (below).

The EOG signals were recorded with two-channel at a 500 Hz sampling frequency, channel 1 refers to horizontal eye movements and channel 2 refers to vertical eye movements. The frequency of the EOG signals was low. Therefore, the recorded signals were downsampled to 20 Hz. to reduce the processing load. Then, the amplitude normalization process was implemented. For amplitude normalization, EOG data was assigned between 0–1 values. In the normalized data, EOG signals were divided into 4 sections including right/left eye movement directions as horizontal eye movements, and also up/down eye movements as vertical eye movement directions. The EOG signals in each direction were divided into 12 sections right, left, up, and down for the evaluation of tracking performance for each part of the three-stage test s indicated in the visual stimulus tracking test. The reference calibration signals were generated to evaluate the follow-up performance of the entire visual stimulus tracking test using the EOG signals. These reference signals were compared with EOG signals recorded during the visual stimulus tracking test from healthy children and patients with ADHD as seen in Figure 2. To obtain information about the number of movements of the eye moving in different directions and for the feature extraction from the EOG signal, signal processing was performed. Firstly, Singular Spectrum Analysis (SSA) was used to eliminate noise components [24,25].

The obtained feature used in this study is the number of one-to-one tracking of the stimuli during the visual stimulus tracking test. In addition to this feature, the colour game detection parameter was used and this parameter is explained in detail below. Also, the EOG signals were analyzed using the Higuchi fractal dimension method for complexity measures and Hjorth parameters for statistical properties. The number of one-to-one tracking of the stimuli, the Colour Game Detection parameter, the Higuchi Fractal Dimension, and the Hjorth parameters obtained from EOG signals were used in the classification study.

##### 2.3.3.1. Higuchi’s fractal dimension (HFD)

Higuchi’s fractal dimension (HFD) is a nonlinear method for the analysis of biological signals [26]. Higuchi’s algorithm calculates fractal dimension (FD) directly from time series. The HFD has been used to analyze the complexity of biological signals [27]. In this study, HFD was used to obtain the complexity of the EOG signals recorded from healthy subjects and patients with ADHD.

##### 2.3.3.2. Hjorth parameters

Hjorth developed a quantitative method to conduct a continuous analysis of physiological signal activity. The three parameters proposed by Hjorth: activity, mobility, and complexity. The signal power, or variance of a time function, is represented by the activity parameter. The mobility parameter measures the power spectrum’s mean frequency or proportion of standard deviation. The frequency change is represented by the complexity parameter [28]. In the proposed study, Hjorth parameters were used to obtain statistical features of the EOG signals recorded from healthy subjects and patients with ADHD.

##### 2.3.3.3. Classification methods

Machine learning algorithms are used to assign a class label with examples from the research/problem domain. In this study, patients with ADHD and healthy children were classified using extracted features from EOG signals with Machine learning algorithms.

Decision tree (DT), support vector machine (SVM), and k-nearest neighbor (KNN) were implemented as machine learning algorithms in order to classify children with ADHD and also healthy subjects [29]. Also, the classification performance of these algorithms was compared.

### 2.4. Parameters used in analysis

#### 2.4.1 Reference stimulus one-to-one tracking numbers (RSOT)

In the visual stimulus tracking test, the number of tracking stimuli appearing at different points and the occurring time of stimuli are determined.

Step 1: In the visual stimulus tracking test, we determined when and where all visual stimuli occur.
Step 2: The time and position of each eye movement in the records obtained from participants were determined.
Step 3: The data obtained in step 1 and step 2 were compared.

Finally, the success percentages of all measurements were calculated. The percentages of success in left-right direction movements were averaged and multiplied by 16. The percentages of success in up-down movements were also averaged and multiplied by 9. A total of 25 divided and weighted success percentages were calculated using the equation below:


$$\text{Weighted success percentage}=(({\left((\text{Left}+\text{right percent success})/2\right)}^{★}16)+{\left((\text{Up}+\text{down percent success})/2\right)}^{★}9))/25$$

#### 2.4.2 Colour game detection (CGD)

There is a relation between stimulus colors and background color in the visual stimulus tracking test. In repeated tests, the background color was the color of the stimulus in the next period. The participants were asked if they realized there was a pattern between the background and the stimulus and their responses were evaluated as true or false.

#### 2.4.3 HFD and Hjorth parameters

HFD and Hjort parameters (activity, mobility, complexity) were calculated for all EOG signals obtained from patients and healthy individuals. The findings were used in the classification study.

### 2.5. Classification

Obtained EOG signal features from patients with ADHD before and after treatment and also HCs were applied to machine learning algorithms for classification. The classification process was performed using the Matlab software package. Classification success of SVM, DT, and KNN algorithms were obtained using extracted features. Data obtained from RSOT, CGD, HFD, and Hjort parameters were used as input data in the classification. The distinction between patients healthy subjects was provided as well as drug-free/after medication.

Obtained data were analyzed by the Statistical Package for Social Sciences (SPSS) for Windows version 22 (IBM SPSS Inc., IL, USA) program. After examining the distribution of the data with the Shapiro–Wilk test, the Independent Sample t-Test, and ANOVA were used to compare the continuous variables with normal distribution within the group, and the Mann-Whitney U test was used to compare the variables that were not normally distributed. The Pearson chi-square test and Fisher’s exact test were used to compare categorical data. Bonferroni correction was used for post hoc analyzes. The statistical significance level was taken as p < 0.05 for all analyses.

## 3. Results

### 3.1. Reference stimulus one-to-one tracking numbers (RSOT)

In Table 1, one to one tracking analysis reports are shown from the first time EOG signal recordings from the drug-free patients, and the second EOG signal recordings after the treatment. One-to-one (by time and location) stimulus tracking accuracy is shown in Table 2 for the groups. The overall performance of HCs was higher than the patients before or after treatment (p < 0.05).

The test performed during obtaining the EOG signals was performed on 16:9 monitors. Because of this screen size, horizontal eye movements were detected more effectively and vertical eye movements were detected in smaller amplitudes. In this context, while determining the overall success percentage of the RSOT parameter, the data for horizontal movements were weighted with 16 and the data for vertical movements were weighted with 9, as shown in Table 3. The difference between groups remained after correction.

### 3.2. Colour game detection (CGD)

The practitioner asked all participants at the end of the test whether there was a relevance between the colors of the visual stimuli and the background colors. Children who explained the pattern correctly were accepted as successful. Questions that could not be answered completely were considered unsuccessful. The CGD success rate was 39.02% in HCs, 45.00% in treated patients, and 4.62% in medication-free patients (for all p < 0.05) (Table 3).

### 3.3. HFD and Hjorth parameters

HFD and Hjorth parameters were calculated for all parts of the test in all patients and HCs using EOG records (horizontal and vertical directions). The average of all the data obtained as a result of the calculation is presented in Table 4.

All the data obtained (i.e. RSOT, CGD, HFD, and Hjort parameters) was used for classification. Tables 5 shows the accuracy rates obtained in the classification performed within the scope of the machine learning algorithms. As a result of classifications, the proposed system distinguished the drug-free ADHD patients from HCs with an 89.13% success rate of classification performance and also distinguished the pretreatment patients with ADHD from their post--treatment status (all responders to treatment) with an 80.47% success rate of classification performance. There was no significant difference between the records of HCs and patients with ADHD after treatment (p > 0.05) (Table 5).

## 4. Discussion

This study introduces a system to assist the diagnosis and follow-up treatment of ADHD with an EOG-based objective approach.

In the previous literature, eye tracking and EOG were investigated in the field of ADHD and other psychiatric disorders. Although there is no study in the literature on the diagnostic support system for ADHD, which uses the stimulus test, Table 6 provides an overview of the current literature. Some previous studies focused on the detection of eye movements and employed an image processing method based on the eye-tracking principle [13–16]. Circular Hough Transform, which aims to recognize circular patterns in an image, was used to detect the iris, and eye movements in video frames [13]. In one study, the background was eliminated by subtracting the face region from the entire image to detect the eye, and then the estimation of the eye region was performed with a Kallman filter. In this way, a method has been developed for eye detection and tracking [14]. Video-based eye-tracking data was used to create a biometric identification model. Obtained features were classified using Back-propagation (BP) neural network and support vector machine (SVM). The results have shown that eye-tracking data can be used for biometric identification [15]. To overcome the limitations of video-based eye-tracking systems in environments where lighting conditions are inadequate, an efficient method of tracking a human eye between consecutively produced infrared interlaced image frames has been developed [16].

Studies also focused on the distinction of ADHD from healthy comparisons, based on the eye-tracking principle. In these studies, the eye movements of individuals in different situations were analyzed [17–20]. The deficits in gaze perception and emotional value judgment during the video-based saccadic eye movement task were investigated in adults with ADHD. It has been suggested that this approach can be used to distinguish adults with ADHD from healthy adults [17]. In a study by Tsang et al. [18], eye-tracking data (image-based) was collected while three participants were watching a video with the scenario, including one child with autism spectrum disorder (ASD), one with ADHD, and one neurotypical control. Differences between participants in social attention were investigated. In another study by Marotta et al. [19], healthy subjects and children with ADHD were asked to categorize target words (left/right), being distracted by arrows or faces that would appear with the words. Differences in arrow interference were identified between subjects with ADHD and healthy participants. In another study, a data set was created by monitoring eye pupil size during a task, in a sample including children diagnosed with ADHD and the control group. This dataset can be used to investigate dynamic pupil and eye movement changes as a function of known behavioral changes and neuropsychological test scores, which indicate neurocognitive processing [20].

On the other hand, in some studies, the classification of ADHD and healthy subjects was performed using a classical statistical approach [21,22]. In the study by Esas et al. [21], EOG signal recordings were obtained from patients with ADHD and healthy children via a visual attention test. The records of patients and healthy subjects were statistically divergent. Latifoğlu et al. [22] developed an EOG recording system with a visual stimulus tracking test. In the developed system, EOG signals were obtained from patients with ADHD and healthy controls, and the signals were analyzed. A significant difference was also observed between ADHD and healthy participants [22].

The findings of the present study showed promising results as an objective approach. When the RSOT, CGD, HFD, and Hjorth parameters were evaluated together, HCs showed a higher success rate than patients. In addition, the test performance increased considerably after treatment, even at higher rates than those seen in HCs. Therefore, clinicians also could use this system to assess the treatment response.

Considering the results of our study using machine learning algorithms, the new system can be used with different features to increase the classification performance for unmedicated patients with ADHD. One-to-one (by time and location) and stimulus tracking accuracy percentages also could support the diagnosis of ADHD. The activity results obtained from HFD and Hijorth parameters could be used as a diagnostic parameter in ADHD after taking into account more parameters obtained from patients.

## Figures and Tables

**Table 1:** Reference stimulus one to one tracking analysis.

Directions	ADHD (Pretreatment) Average	ADHD (Pretreatment) Success Rate	ADHD (Posttreatment) Average	ADHD (Posttreatment) Success Rate	HCs average	HCs success rate	Reference
**Left-1**	13.05	32.63%	13.36	33.40%	15	37.50%	40
**Left-2**	11.87	29.68%	13.51	33.78%	14.41	36.02%	40
**Left-3**	13.15	32.88%	12.77	31.92%	14.31	35.78%	40
**Right-1**	10.97	27.44%	11.28	28.21%	12.84	32.11%	40
**Right -2**	9.87	24.68%	11.1	27.76%	12.06	30.16%	40
**Right -3**	10.03	25.06%	10.13	25.32%	11.06	27.66%	40
**Up-1**	7.23	24.10%	7.82	26.07%	8.06	26.88%	30
**Up -2**	8.67	28.89%	8.49	28.29%	10.28	34.27%	30
**Up -3**	8.64	28.80%	8.54	28.46%	9.84	32.81%	30
**Down-1**	6.72	22.39%	8.08	26.92%	7.5	25.00%	30
**Down -2**	8.03	26.75%	7.9	26.32%	9.56	31.88%	30
**Down -3**	9.15	30.51%	8.08	26.92%	10.56	35.21%	30

Attention deficit hyperactivity disorder (ADHD), healthy controls (HCs)

**Table 2:** Stimulus tracking accuracy rate.

Directions	ADHD (Pretreatment)	ADHD (Posttreatment)	HCs
**Left**	31.73%	33.03%	36.43%
**Right**	25.73%	27.10%	29.98%
**Up**	27.26%	27.61%	31.32%
**Down**	26.55%	26.72%	30.70%
**Average**	**27.82%**	**28.62%**	**32.10%**

**Table 3:** RSOT weighted average and CDG success rate.

	ADHD (Pretreatment)	ADHD (Posttreatment)	HCs
**RSOT**	**28.07%**	**29.02%**	**32.41%**
**CGD**	**4.62%**	**45.00%**	**39.02%**

Reference stimulus one-to-one tracking numbers (RSOT), Colour game detection (CGD)

**Table 4:** Average results obtained from HFD and Hjorth parameters.

Average	First part				Second part				Third part			
	HFD	Activity	Mobility	Complexity	HFD	Activity	Mobility	Complexity	HFD	Activity	Mobility	Complexity
**ADHD (Pretreatment) EOG**	1.056	0.0728	0.0151	0.3789	1.0533	0.0878	0.0145	0.4476	1.053	0.0991	0.0137	0.4967
**ADHD** **(Posttreatment) EOG**	1.052	0.0832	0.0143	0.3602	1.0501	0.1009	0.0137	0.3855	1.050	0.1218	0.0132	0.4970
**HCs EOG**	1.051	0.1321	0.0149	0.3112	1.0495	0.1495	0.0148	0.4588	1.049	0.1209	0.0142	0.4901

Higuchi’s Fractal Dimension (HFD)

**Table 5:** Average classification accuracy according to algorithms.

**ADHD (Pretreatment) /HCs classification accuracy results**				
	Machine Learning Algorithms	Cross-Validation Folds = 5	Cross-Validation Folds = 10	No validation
	DT	66.27%	62.50%	**89.13%**
	SVM	59.02%	56.25%	86.90%
	k-NN	55.07%	50.00%	73.97%
**ADHD (Pretreatment) /ADHD(Posttreatment)/HCs classification accuracy results**				
	Machine Learning Algorithms	Cross-Validation Folds = 5	Cross-Validation Folds = 10	No validation
	DT	43.77%	58.37%	**80.47%**
	SVM	43.22%	58.33%	80.07%
	k-NN	35.30%	30.55%	66.13%

Decision Tree (DT), Support Vector Machine (SVM), and k-Nearest Neighbor (KNN)

**Table 6:** The overview of the previous literature on eye movements in ADHD.

Study	Data type	Method	Sample	Procedures and findings
(Al-Rahayfeh, et al., 2013)	Video image	Eye-tracking, Image Processing	Adults	**A real-time eye-tracking system using Circular Hough Transform for eye detection** was proposed. Detection of eye-gaze, shortened central processing unit processing time. The required processing time for low-speed eye motion was improved by a factor of 1500% and for high-speed eye movement was 750%.
(Kashani, et al., 2011)	Video image	Eye-tracking, Image Processing	6 adults	It was suggested a new method that **detects the position of the pupil** with high accuracy. Eye detection and tracking were applied on test sets collected from different images of face data with complex backgrounds. Detection performance 94.9%.
(Liang, et al., 2012)	Video image	Eye-racking, Image Processing	5 adults	**A biometric identification model** based on high-accuracy video-based eye tracking has been proposed using machine learning algorithms. Video clips were used to capture eye-tracking data reflecting the subjects’ physiological and behavioral characteristics. Detection performance 82%.
(Hansen, et al., 2007)	Video image	Eye-tracking, Image Processing	Adults	This study offered a **reliable eye-tracking method between successively produced infrared interlaced image frames considering the lighting conditions**. Detection performance 91.17%. The classification was made with and without the use of dark and bright pupil images. It was found that the overall detection rate was decreased when using the dark and bright pupil differences as verification.
(Lee, et al., 2015)	Saccadic eye movement task	Gaze-emotion recognition	16 ADHD and 16 healthy subjects were recruited from 243 adult students (mean age 21)	**Classification of ADHD and healthy subject** If the cue face was not emotionally neutral, the participant’s responsibility was to perform an antisaccade in the opposite direction of the gaze direction. In making antisaccades, ADHD patients experienced more overall errors than controls. Detection performance p < 0.05. It has been demonstrated that ADHD patients and healthy adults can be classified according to the deficits in gaze perception during the video-based saccadic eye movement task.
(Tsang, et al., 2018)	Video image	Eye-tracking	1 ASD, 1 ASD+ADHD, and 1 healthy subject,	**Classification of ASD, ASD+ADHD, and healthy subject**For the three children, each child watched the same video about one min long, that consisted of daily life scenarios. Performance: Visual attention was reduced in children with ADHD and that social fixation duration was shorter than healthy control
(Marotta, et al. 2017)	Video image	Data processing, feature extraction	19 ADHD, and 19 healthy subjects aged 7–17 years	**Classification of ADHD and healthy subjects** Performance: Healthy and children with ADHD were asked to categorize target words (left/right) accompanied by distracting arrows or faces that would appear with the words. Differences in arrow interference were identified between subjects with ADHD and healthy participants
(Rojas-Líbano, et al. 2019)	Video image	Eye-tracking	28 ADHD and 22 healthy subjects; aged 8–13	**Classification of ADHD and healthy subjects** Performance: It was evaluated that the findings obtained from the study could distinguish ADHD/healthy. The data set was shared which can be used to investigate dynamic pupil and eye movement changes as a function of known behavioral changes and neuropsychological test scores
(Esas, et al. 2017)	EOG Signals	Eye-tracking	8 children with ADHD and 8 Healthy Subjects	**Classification of ADHD and healthy subject** The reference directions on the test were calculated using attention test software that provided an analysis of eye movements during signal processing. The amount of eye movement in the participants who underwent the EOG attention test was found to be more compatible with the test in healthy subjects. Detection performance p < 0.05
(Latifoglu, et al. 2020)	EOG Signals	Eye-tracking	8 children with ADHD and 8 Healthy Subjects; aged 8–13	**Classification of ADHD and healthy subject** According to the results of the t-test, no significant difference was found (p = 0.11) between the healthy group and the reference movement information, whereas a significant difference was found between patients and the reference motion information (p = 0.049). Performance: The mean accuracy rate was 93.11% in healthy subjects and 85.14% in patients.

Autism spectrum disorder (ASD), attention deficit-hyperactive disorder (ADHD)
